# Effect of Tactile Stimulation on Termination and Prevention of Apnea of Prematurity: A Systematic Review

**DOI:** 10.3389/fped.2018.00045

**Published:** 2018-03-02

**Authors:** Sophie J. E. Cramer, Janneke Dekker, Jenny Dankelman, Steffen C. Pauws, Stuart B. Hooper, Arjan B. te Pas

**Affiliations:** ^1^Department of Instrumental Affairs, Leiden University Medical Center, Leiden, Netherlands; ^2^Division of Neonatology, Department of Pediatrics, Leiden University Medical Center, Leiden, Netherlands; ^3^Department of Biomedical Engineering, Delft University of Technology, Delft, Netherlands; ^4^Tilburg center for Cognition and Communication (TiCC), Tilburg University, Tilburg, Netherlands; ^5^The Ritchie Center, MIMR-PHI Institute of Medical Research, Melbourne, VIC, Australia

**Keywords:** preterm infants, tactile stimulation, apnea of prematurity, apnea, breathing

## Abstract

Apnea of prematurity (AOP) is one of the most common diagnoses in preterm infants. Severe and recurrent apneas are associated with cerebral injury and adverse neurodevelopmental outcome. Despite pharmacotherapy and respiratory support to prevent apneas, a proportion of infants continue to have apneas and often need tactile stimulation, mask, and bag ventilation and/or extra oxygen. The duration of the apnea and the concomitant hypoxia and bradycardia depends on the response time of the nurse. We systematically reviewed the literature with the aim of providing an overview of what is known about the effect of manual and mechanical tactile stimulation on AOP. Tactile stimulation, manual or mechanical, has been shown to shorten the duration of apnea, hypoxia, and or bradycardia or even prevent an apnea. Automated stimulation, using closed-loop pulsating or vibrating systems, has been shown to be effective in terminating apneas, but data are scarce. Several studies used continuous mechanical stimulation, with pulsating, vibrating, or oscillating stimuli, to prevent apneas, but the reported effect varied. More studies are needed to confirm whether automated stimulation using a closed loop is more effective than manual stimulation, how and where the automated stimulation should be performed and the potential side effects.

## Introduction

Almost all infants born at <28 weeks gestational age (GA) or with a birth weight of <1,000 g are diagnosed with apnea of prematurity (AOP) (1). The American Academy of Pediatrics defines apnea as a cessation of breathing for 20 s or a shorter pause accompanied by bradycardia, cyanosis, or pallor (2). Based on their origin, apneic spells are classified as central, obstructive, or mixed. Central apnea is distinguished by a cessation of airflow due to absence of respiratory drive, obstructive apnea is characterized by impeded airflow caused by closure of the upper airways and mixed apnea implies that central respiratory pauses are followed by obstruction in the upper airways or vice versa (3–5). Studies have shown that most of the apneas in a preterm infant have a mixed character, starting with a central or an obstructive episode (6).

The etiology is related to the immaturity of respiratory control and poor myelination of the brainstem (5, 7) but the exact responsible mechanisms are still not fully understood (5, 8). Although apnea generally resolves with maturation, it is one of the most common diagnoses and therefore a major concern in the Neonatal Intensive Care Unit (NICU) (4, 8, 9). Frequent apneic spells can lead to serious cerebral injury and affects neurodevelopmental outcome (10–12). It has been postulated that the adverse outcome is not caused by the apnea itself but the associated recurrent hypoxia (4, 9, 13).

In most NICUs both pharmacotherapy and breathing support are used to prevent recurrent AOP. Despite these preventative interventions, a proportion of infants continue to have apnea (14), which requires further intervention of the caregiver. The termination of apnea is accomplished by tactile intervention of the nurse, often combined with extra oxygen and, if needed, mask ventilation (6, 15–17). The duration of the apnea and the concomitant hypoxia and/or bradycardia is then dependent on the response time of the nurse. Heavy workload and alarm fatigue might have a negative influence on prompt and adequate treatment of apneas (18). The longer the delay in response time, the longer the total duration of apnea and the lower the peripheral oxygen saturation (SpO_2_) values (19). Also, administration of tactile stimulation increases the risks of infection due to cross-contamination and will interrupt sleep, which can be disadvantageous for the growth and development of the infant (20).

Mechanical stimulation might improve the common used and effective tactile stimulation technique by enabling a direct response, as this will shorten the apnea hence reducing hypoxia and bradycardia. In addition, combining mechanical stimulation with the detection of imminent apnea could lead to preventive stimulation methods. The effect of mechanical tactile stimulation on apnea has been studied but has not led to implementation in the NICU or a commercially available device yet.

We systematically reviewed the literature with the aim of providing an overview of what is known about the effects of manual and mechanical tactile stimulation on the termination and prevention of apnea in preterm infants.

## Methods

To identify convenient studies the online databases MEDLINE, PubMed, and Scopus were searched for English articles from 1970 to 2017, using the search strategy as described in Table 1. The time span was based on the results of a Cochrane review of kinesthetic stimulation to treat AOP (21). A manual search of the references and citations of the selected articles was performed to collect other possible relevant literature. Unpublished data were not considered for this review.

**Table 1:** Search strategy.

Database	Keywords	Hits
Medline	(touch OR touching OR touches OR touched OR rub OR rubbing OR rubbed OR scratch OR scratched OR scratching OR cutaneous OR skin OR mechanosensory OR vibration OR vibrating OR vibratory OR vibrotactile OR foot OR feet OR sole OR back OR thorax OR arousal OR stochastic resonance).ti,ab. AND (vibration OR vibrations OR vibratory OR vibrate OR vibrates OR vibrated OR physical stimulation OR stimulation OR stimulations OR stimulate OR stimulates OR stimulated OR stimulus OR stimuli OR system).ti,ab. AND (apnea OR apnoea OR breathing OR breath OR breathe OR breathes OR breathed).ti,ab. AND (premature OR prematures OR prematurity OR preterm OR preterms OR neonate OR neonates OR neonatal OR infant OR infants).ti,ab. AND (treat OR treatment OR treating OR treated OR interrupt OR interruption OR interrupting OR interrupted OR stabilize OR stabilizing OR stabilized OR analyze OR analysis OR analyzing OR analyzed OR transform OR transformation OR transforming OR transformed OR generate OR generation OR generating OR generated OR effect OR effects OR effecting OR effected).ti.ab.	105

PubMed	(“touch”[mesh] OR “touch”[tw] OR “touching”[tw] OR “touches”[tw] OR “touched”[tw] OR “rub” [tw] OR “rubbing” [tw] OR “rubbed” [tw] OR “scratch” [tw] OR “scratched” [tw] OR “scratching” [tw] OR “cutaneous”[tw] OR “skin”[tw] OR “mechanosensory”[tw] OR “vibration” [tw] OR “vibrating” [tw] OR “vibratory” [tw] OR “vibrotactile” [tw] OR “foot”[mesh] OR “foot”[tw] OR “feet”[tw] OR “sole”[tw] OR “back” [tw] OR “thorax” [tw] OR “arousal”[mesh] OR “arousal”[tw] OR “stochastic resonance” [tw]) AND (“vibration”[mesh] OR “vibration”[tw] OR “vibrations”[tw] OR “vibratory”[tw] OR “vibrate”[tw] OR “vibrates”[tw] OR “vibrated”[tw] OR “physical stimulation”[mesh] OR “stimulation”[tw] OR “stimulations”[tw] OR “stimulate”[tw] OR “stimulates”[tw] OR “stimulated”[tw] OR “stimulus”[tw] OR “stimuli”[tw] OR “system”[tw]) AND (“apnea”[mesh] OR “apnea”[tw] OR “apnoea”[tw] OR “breathing”[tw] OR “breath”[tw] OR “breathe”[tw] OR “breathes”[tw] OR “breathed”[tw]) AND (“infant, premature”[mesh] OR “premature”[tw] OR “prematures”[tw] OR “prematurity”[tw] OR “preterm”[tw] OR “preterms”[tw] OR “neonate”[tw] OR “neonates”[tw] OR “neonatal”[tw] OR “infant”[tw] OR “infants”[tw]) AND (“treat”[tw] OR “treatment”[tw] OR “treating”[tw] OR “treated”[tw] OR “interrupt”[tw] OR “interruption”[tw] OR “interrupting”[tw] OR “interrupted”[tw] OR “stabilize”[tw] OR “stabilization” [tw] OR “stabilizing[tw] “ OR “stabilized”[tw] OR “analyze”[tw] OR “analysis”[tw] OR “analyzing”[tw] OR “analyzed”[tw] OR “transform”[tw] OR “transformation”[tw] OR “transforming”[tw] OR “transformed”[tw] OR “generate”[tw] OR “generation”[tw] OR “generating”[tw] OR “generated”[tw] OR “effect”[tw] OR “effects”[tw] OR “effecting”[tw] OR “effected”[tw])	190

Scopus	TITLE-ABS(“touch” OR “touching” OR “touches” OR “touched” OR “rub” OR “rubbing” OR “rubbed” OR “scratch” OR “scratched” OR “scratching” OR “cutaneous” OR “skin” OR “mechanosensory” OR “vibration” OR “vibrating” OR “vibratory” OR “vibrotactile” OR “foot” OR “feet” OR “sole” OR “back” OR “thorax” OR “arousal” OR “stochastic resonance”) AND TITLE-ABS (“vibration” OR “vibrations” OR “vibratory” OR “vibrate” OR “vibrates” OR “vibrated” OR “physical stimulation” OR “stimulation” OR “stimulations” OR “stimulate” OR “stimulates” OR “stimulated” OR “stimulus” OR “stimuli” OR “system”) AND TITLE-ABS (“apnea” OR “apnoea” OR “breathing” OR “breath” OR “breathe” OR “breathes” OR “breathed”) AND TITLE-ABS (“premature” OR “prematures” OR “prematurity” OR “preterm” OR “preterms” OR “neonate” OR “neonates” OR “neonatal” OR “infant” OR “infants”) AND TITLE-ABS (“treat” OR “treatment” OR “treating” OR “treated” OR “interrupt” OR “interruption” OR “interrupting” OR “interrupted” OR “stabilize” OR “stabilizing” OR “stabilized” OR “analyze” OR “analysis” OR “analyzing” OR “analyzed” OR “transform” OR “transformation” OR “transforming” OR “transformed” OR “generate” OR “generation” OR “generating” OR “generated” OR “effect” OR “effects” OR “effecting” OR “effected”)	153

All clinical trials reporting the effects of tactile stimuli on apnea in premature infants or animals were included in this review. Studies using devices that are believed to affect the breathing patterns by other forms of stimulation that involved a tactile component, like oscillating waterbeds, were included. Clinical trials examining the effect of stimulation of multiple senses on apnea were excluded. The same applied to articles comparing only the effects of tactile stimulation with stimulation of another sense. Abstracts or other forms of articles that are not primary research studies were also excluded. Two authors (Sophie J. E. Cramer and Arjan B. te Pas) reviewed the records for inclusion and exclusion criteria, and disagreements were resolved by consensus.

Study characteristics from the included studies were identified using a data extraction form. The following data were extracted: author, year, study objects, study design, detection signals, stimulation mode, stimulation characteristics, duration, and main results.

## Results

**Figure 1:**
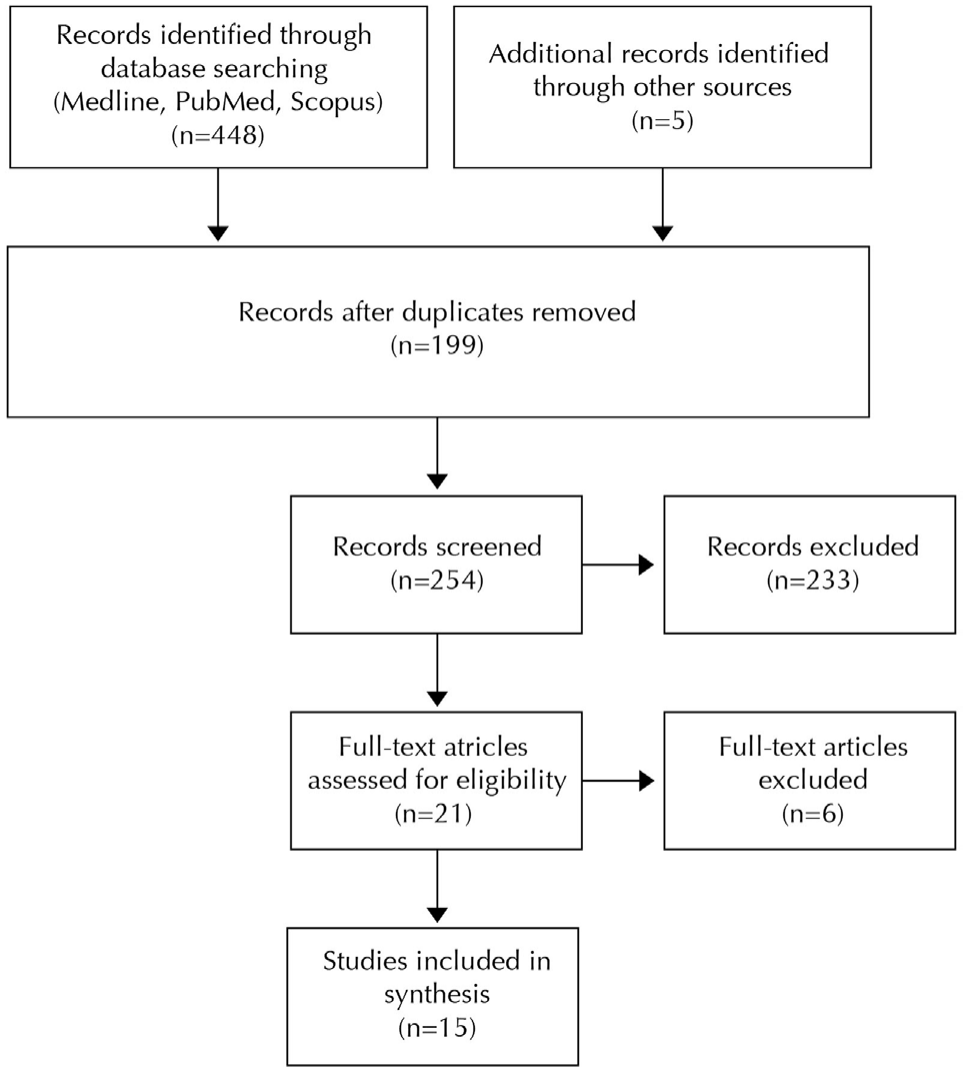
Flowchart of article selection.

The search strategy led to 448 articles. Five additional articles were selected from the references of the studies that met the inclusion criteria. After elimination of the duplicates, a selection of 21 articles was made based on title and abstract. Another 6 articles were excluded following full-text assessment, resulting in a selection of 15 articles for this review (Figure 1). Four of these studies investigated the effect of tactile stimulation on the termination of apnea, and 11 focused on the effect on the prevention of apnea.

Combining the data of the studies for a meta-analysis was not possible since there was no homogeneity in study designs, study objects, mode of stimulation, and measure for effect size. For this reason the results were reviewed in a narrative format, where we report separately for the terminating and the preventing apneas. The extracted data of the articles are summarized in Tables 2 and 3.

**Table 2:** Termination of apnea.

Reference/study design	Study objects	Detection signals	Stimulation characteristics	DurationStimulation time	Results of stimulation
Camargo et al. (22) Observational study	4 infants Gestational age (GA) inclusion: <36.6 weeks Weight inclusion: <2,500 g	Pulse frequency by oximeter Peripheral oxygen saturation (SpO_2_) by oximeter	Instrument: VBW32 skin transducer, audiological engineering Location: thorax Stimulus: 4 s, 250 Hz	Stimulation: 4 s	**Apnea (HR < 100 bpm, SpO_2_ < 80% for <35 weeks, HR < 80 bpm, SpO_2_ < 80% for >35 weeks):** Resumption of breathing in 9 of 10 apneic episodes

Pichardo et al. (19) Randomized crossover study	4 infants GA inclusion: >28 weeks Weight inclusion: >1,000 g	Nasal airflow (AF) HR by chest leads and saturation probe SpO_2_ Electrocardiogram Thoracic impedance	*Mechanical* Instrument: VTS transducer VBW32, audiologic engineering Location: thorax Stimulus: not described *Manual* No specifications	Stimulation: 3 s Mode switch: after 12 h or after 5 apneic attacks Duration: 24 h Repetitions: 2× for one infants	**Apnea:** Similar effectiveness in ending apneic spells (*p* = 0.62) Significant decrease in stimulus duration (*p* < 0.001) Decrease in reaction time to apneic spell (*p* = 0.058)

Lovell et al. (23) Randomized crossover study	1 infant GA: 28 weeks Weight: 1,280 g Day start study: 7 Treatment: aminophylline, first 2 days on continuous positive airway pressure	Respiration rate HR SpO_2_	*Mechanical* Instrument: model 1220 audiological engineering Location: sole of the foot Stimulus: 250 Hz square wave, impedance of 22 Ω (vts) *Manual* Location: trunk and extremities Stimulus: gentle to vigorous stroking or shaking of the trunk and/or extremities (manual)	Stimulation: approximately 3 s Mode switch: after 8 h Duration: 16 h Repetitions: three times after 2, 6, and 11 days	**Apnea (>20 s or <20 s + bradycardia/intermittent hypoxia):** Similar effectiveness in ending apneic spells (*p* > 0.05) Decrease in stimulation time of 3.9 s (*p* = 0.05) Similar reaction time (*p* = 0.93) Similar time to termination (*p* = 0.67) Similar apnea duration (*p* = 0.94)

Frank et al. (24) Observational study	4 infants GA: 31.25 weeks Weight: 1,495.5 g All infants asleep	Respiration rate by impedance plethysmography	Instrument: balloon with a towel. Location: transversely under the neck Stimulus: 4 psi pressure source, inflation in 0.5 s	Stimulation: 3 pulses	**Apnea (10 s, sometimes 5 or 15 s):** Resumption of breathing in 99 of the 105 apneic episodes

**Table 3:** Prevention of apnea.

Reference/study design	Study objects	Detection signals	Stimulation characteristics	DurationStimulation time	Results of stimulation
Kesavan et al. (25) Randomized crossover study	15 infants Gestational age (GA): 29.0 ± 2.5 weeks Weight: 1,257 ± 535 g Study age: 32 ± 2.3 weeks Treatment: 12 caffeine, 12 supplemental oxygen	Respiratory movement (RM) by thoracic wall movement measurement heart rate (HR) *via* 3 leads Peripheral oxygen saturation (SpO_2_) by pulse oximetry Electrocardiogram (ECG) signals	Instrument: vibrating disk (10 mm × 10 mm × 3 mm) connected to vibration motor Location: palm or wrist of one hand and the ankle or sole of the foot at the same side Stimulus: 0.3 g/128 Hz vibration	Stimulation: 6 h alternatively on or off Duration: 24 h	**Breathing pauses—significant reduction of:** Amount >5 s by 39% (*p* < 0.001); Amount 3–5 s by 21% (*p* = 0.024) Duration >5 s by 36% (*p* < 0.001); Duration 3–5 s by 20% (*p* = 0.034) **Intermittent hypoxia (IH)—significant reduction of:** Amount <90% by 28% (*p* = 0.001); <88% (*p* = 0.001); <85% (*p* < 0.001) Duration <90% by 30% (*p* = 0.002); <88% (≤0.001); <85% (*p* = 0.023) **Bradycardia—significant reduction of:** Amount <110 bpm (*p* = 0.001); <100 bpm (*p* = 0.002) Duration <110 bpm (*p* = 0.003); <100 bpm (*p* = 0.006)

Smith et al. (14) Randomized crossover study	36 infants GA: 30.5 ± 2.9 weeks Weight: 1,409 ± 450 g Study age: 35.0 ± 1.5 Study weight: 2,013 ± 453 Treatment: 13 caffeine, 5 supplemental oxygen	Respiration rate (RR) by VueLogger system HR SpO_2_ ECG Pulse plethysmography	Instrument: 13 TheraSound mattress, 23 custom build mattresses Location: not described Stimulus: displacements with frequency bandwidth of 30–60 Hz and displacements of 10–20 µm	Stimulation: 30 min alternately on or off Duration: 3–4 h	**Breathing pauses:** Reduction amount of pauses >10 s by 50% **IH (<87% for >35 weeks GA, <90% for <35 weeks GA)—significant reduction of:** Amount by 18% (*p* = 0.01), duration by 35% (*p* < 0.0001), intensity by 21% (*p* < 0.0001) **Bradycardia (<80 bpm for >34 weeks GA, <100 bpm for <34 weeks):** No change in amount and duration of bradycardia Significant reduction intensity of bradycardia of 20% (<0.0001) **Other:** No significant effect of supplemental oxygen, caffeine, pma, and weight. Significant effect of light on duration and intensity IH Significant effect of sound on duration of IH

Bloch-Salisbury et al. (26) Randomized crossover study	10 infants GA: 30.1 ± 1.9 weeks Weight: 1,348 ± 497 g Study age: 33.1 ± 1.7 weeks Study weight: 1,500 ± 441 g Treatment: 1 nasal cannalue oxygen, 3 caffeine before test	RM by respiratory inductance plethysmography AF and CO_2_ by thermistor or cannalue Pulse frequency by pulse oximeter SpO_2_ by pulse oximeter Skin temperature by temperature probe	Instrument: therasound mattresses Location: chest, side, or back, depending on position Stimulus: filtered white noise, 30–60 Hz band, stimulus intensity of 0.021 mm RMS, 0.090 mm max displacement	Stimulation: 10 min alternately on or off Duration: 1 h Repetitions: 8 of 10 infants completed the experiment twice	**Interbreath intervals—a significant reduction of:** Variance IBIs (*p* = 0.024) Amount IBIs; >5 s (*p* = 0.013); >10 s (*p* = 0.042) **IH—a significant reduction of:** Amount of time O_2_ < 85% (*p* = 0.04) **Bradycardia:** Reduction in pulse rate variance (*p* = 0.086) Mean pulse rate was unaffected by stimulation (*p* = 0.14) **Other:** No significant changes in behavioral state or electroencephalogram power spectra during stimulation transitions No significant changes in skin temperature

Svenningsen et al. (32) Quasi-experimental crossover study	12 infants GA: 31.1 weeks Weight: 1.760 g Treatment: 6 theophylline, 4 continuous positive airway pressure (CPAP)	Cardiorespirography and concomitant oxygen	Instrument: OSCILLO-unit (electronic membrane pump with 2 pneumatic valves for in- and outflow of an airfilled mattress) Location: not described Stimulus: oscillation amplitude 10–100%, frequency 5–20 times/min, high frequency vibrations 8–10 Hz	Stimulation: 12 h (24 h for 9 infants) Control: 24 h before stimulation	**Apnea (pause of >20 s + < 85% O_2_ + decrease in hour of >20 bpm):** Mean apneic attacks control period: 8.4 per 12 h Mean apneic attacks first 12 h stimulation period: 3.0 per 12 h Mean apneic attacks second 12 h stimulation period: 3.8 per 12 h **Other:** 3 infants showed restlessness after stimulation 4 infants increased intra-arterial blood pressure around 5 mm Hg

Jirapaet (27) Quasi-experimental counterbalanced crossover study	29 infants GA: 32.1 ± 1.8 weeks Weight: 1,474 ± 331 g Day start study: 4.3 ± 3.0 days Treatment: 2 assisted ventilation	AF by thermistor HR and breathing effort by apnea monitor (model 500, corometrics) SpO_2_ by oximeter	Instrument: blood pressure cuff connected to bird’s mark 8 respirator which in and deflates the cuff Location: under upper thorax Stimulus: in and deflates 16 + 4 times per min, regular vertical wave motion of 1 cm at the cuff surface	Stimulation: 6 h alternatively on or off Duration: 24 h	**Apnea (HR < 100 bpm or pause > 15 s):** Significant reduction of apneic episodes during stimulation (*p* < 0.000) Significant reduction of central apneas (*p* < 0.000) and mixed apneas (*p* < 0.000) No effect on obstructive apneas (*p* = 0.316)

Saigal et al. (33) Controlled randomized clinical trial	122 infants Exp. group (*n* = 59) GA: 30.5 ± 3.2 weeks Weight: 1,294 ± 266 Medicines: 28 theophylline, 20 CPAP/intermittent positive pressure ventilation (IPPV) Control group (*n* = 63) GA: 31.0 ± 2.7 weeks Weight: 1,299 ± 41 Treatment: 22 theophylline, 12 CPAP/IPPV	Cardiorespiratory impedance Cardiorespirograph for 6 h between study days 1–3, 4–7, 8–12, and after 13 days to check nurses administration	*Experimental group* Instrument: oscillating air mattresses Location: not described Stimulus: 14–16 pulses/min, longitudinal wave motions *Control group* Conventional mattress, no stimulation	Stimulation: 7 days or more (until discharge)	**Apnea:** No significant reduction in amount apnea 10–19 s No significant reduction in amount apnea >20 s No significant reduction in amount apnea >10+ bradycardia **Other:** No significant differences in weight gain No significant differences in proportions time in sleep states and state changes

Korner et al. (28) Quasi-experimental counterbalanced crossover study	17 infants GA: 29 weeks Weight: 1,159 g Day start study: 35 days Treatment: all theophylline for 6–71 days	RR HR Visual observations for 100 min on 3rd and 4th day after feeding	Instrument: water bed, consists of high impact styrene shell and vinyl bag with small inflatable bladder at the foot connected to an electronic oscillator. Location: not described Stimulus: continuous gentle irregular head-to-foot oscillations, 8/10 oscillations/min with amplitude of 2.4 mm	Stimulation: 4 days alternatively on or off Duration: 8 days	**Apnea:** No significant reduction in amount of apnea (*p* > 0.05) No significant reduction in amount of apnea with cyanosis (*p* > 0.05) **Bradycardia:** No significant reduction in amount of HR 80–100 bpm (*p* > 0.05) No significant reduction in amount of HR < 80 bpm (*p* > 0.05) **Other:** Significant more quiet sleep, les state changes, less restlessness

Jones (30) Randomized crossover study	14 infants GA (median): 29.4 weeks Weight (median): 1,080 g Study age: Day start study (median): 8 days Study weight: Treatment: 3 theophylline	ECG Impedance pneumogram	*Oscillating* Instrument: same mattress as described by Korner, inflatable bladder under the head end. Location: not described Stimulus: 12–14 oscillations/min, amplitude 1–2 mm. *Non-oscillating*Instrument: same mattress Location: not described Stimulus: none	Stimulation: 4 h alternatively on or off Duration (mean): 23 h Extra: 10 infants another 11 h with the mattress emptied of water, divided between the beginning, middle, and end of the time of the waterbed	**Apnea:** No significant reduction in amount of apnea 3–9 s (*p* > 0.1) No significant reduction in amount of apnea >10 s (*p* > 0.1) Apneas of >10 s happened in 5 out of 6 infants more frequent on the oscillating bed. **Bradycardia:** Significant increase in amount of bradycardia < 60 bpm (*p* < 0.02) No significant reduction in amount of bradycardia <80 bpm (*p* > 0.1) No significant reduction in time of HR < 80 bpm (*p* > 0.1) **Other:** 0.1 C decrease in mean body temperature Hypothermia in 1 infant No significant differences of any of the parameters measured on the waterbed and on the emptied waterbed

Korner et al. (29) Quasi-experimental counterbalanced crossover study	8 infants GA: 30 weeks Weight: 1,270 g Day start study: 15 days Study weight: 1,264 g Treatment: no other than antibiotics	Respiration by mercury-filled strain gauges and a thermistor in front of each nostril Electroencephalogram, electrooculogram, electromyogram, and ECG by electrodes	Instrument: waterbed, Baumanometer blood pressure bladder connected to an Emerson respirator Location: not described Stimulus: gentle irregular head-to-foot oscillations, 12–14 pulses/min, 2.4 mm amplitude	Stimulation: 6 h alternatively on or off Duration: 24 h	**Apnea during sleep on the waterbed:** Decrease in amount of apneas >10 s (*p* < 0.06) Decrease central apneas *p* < 0.10 Decrease obstructive mixed apneas *p* < 0.08 Significant decrease in amount of apnea + HR 80–120 (*p* < 0.02) Significant decrease in amount of apnea + HR < 80 (*p* < 0.05) Apnea during REM increased, during quiet sleep decreased and during intermediate sleep significantly decreased

Korner et al. (34) Controlled randomized clinical trial	21 infants Experimental group (*n* = 10) GA: 32 weeks Weight: 1,521 g Control group (*n* = 11) GA: 31.3 weeks Weight: 1,382 g	HR, RR, temperature, concentrations administered oxygen Apnea by alarm and notes nurse	*Experimental group* Instrument: waterbed, styrene shell with small inflatable rubber bladder connected to Emerson respirator Location: not described Stimulus: irregular head-to-foot oscillation, inflates, and deflates the bag 16 ± 4 times/min *Control group* Instrument: conventional foam-rubber mattress	Stimulation: 7 days	**Apnea (RR < 20 breaths/min and/or HR < 100 bpm):** Significant decrease in amount of apnea (*p* < 0.01) **Other:** No significant changes in apical pulse, respiratory rate, temperature, weight, and emesis

Kattwinkel et al. (31) Quasi-experimental crossover study	6 infants GA: 28 weeks Weight: 1,103 g Day start study: 8	Cardiorespiratory by impedance measurements HR and respiratory pneumograms by dynograph recorder	Instrument: hand Location: extremities Stimulus: rubbing	Stimulation: 5 out of 15 min Duration: 3 h Control: 3 h before stimulation	**Apnea:** Significant decrease in amount of apnea (*p* < 0.01). This difference was present for the entire 3-h test period and also for the 2 h of the test period during which time cutaneous stimulation was not being administered

### Termination of Apnea

Four studies were included that provided tactile stimulation to terminate apnea in 13 preterm infants. The sample size ranged from one to four infants with a median of four infants. The mean GA varied between studies from 28 to 31.25 weeks and the mean birth weight varied from 1,280 to 1,495.5 g. Two studies only reported inclusion criteria instead of mean values for GA and birth weight (19, 22). In one study, aminophylline was administered during the study, which started 7 days after birth (23). Frank et al. only included sleeping infants (24).

#### Study Designs

The included studies described different study designs: two observational (22, 24) and two randomized crossover studies (19, 23). In the observational studies the amount of successfully terminated apneas were compared with the total amount of apneas. In the randomized crossover studies, the infants were stimulated alternately by hand or with an automatic device for a set time. Lovell et al. (23) used periods of 8 h with a total time of 16 h, and Pichardo et al. (19) used periods of 12 h with a total time of 24 h.

#### Stimulation Systems

There was a considerable variation between the studies in the detection of apneas and stimulation systems used. Camargo et al. (22) used heart rate (HR) and oxygen saturation measurements to identify apnea. A decrease in oxygen saturation or HR below the set threshold of 80% and 80 or 100 bpm automatically actuated a vibrating disk attached to the infants’ thorax, which exerts a vibration of 250 Hz for 4 s. Frank et al. (24) also used a closed-loop system. Breathing pauses were identified by impedance plethysmography. Exceeding of the set threshold, ranging from 5 to 15 s, automatically actuated a balloon placed under the neck of the infant, which then inflated and deflated three times. The remaining two studies used similar systems, which were manually actuated by the nurses. Lovell et al. (23) recorded HR and oxygen saturation and used a 3-s vibrating stimulus of 250 Hz at the foot sole. Pichardo et al. (19) additionally recorded airflow, electrocardiogram and thoracic impedance and used the same apparatus with the same stimulus but applied it at the thorax.

#### Effect

The pulsating balloon of Frank et al. placed underneath the neck, led to resumption of breathing in 99 of the 105 detected apneic spells (24). Camargo et al. (20) observed resumption of breathing following vibratory stimulation in 9 of 10 apneas. The other two studies reported that the vibrating stimulation was as effective as manual stimulation in aborting apneic spells (19, 23), but that the duration of the vibratory stimulus was shorter than the manual stimulation. The response time for mechanical stimulation was shorter than for manual stimulation in the study of Pichardo et al. (19) while in the study of Lovell et al., they were of equal duration (23).

### Prevention of Apnea

In total, 11 studies investigated the prevention of apnea by tactile stimulation and included 290 preterm infants. The sample size ranged from 6 to 122 infants with a median of 15 infants. The mean/median GA varied between studies from 28 to 32.1 weeks. Three studies reported the GA at the start of the study, ranging from 32 to 35 weeks (14, 25, 26) and five studies reported the (mean/median) age when the study started, ranging from 4.3 to 35 days after birth (27–31). The mean/median birth weight also differed between studies from 1,080 to 1,760 g. Three studies reported a mean weight of 1,264–2,013 g at the start of the study (14, 26, 29). In a number of studies (some of) the infants were supported by means of: administered caffeine (14, 25, 26), theophylline (28, 30, 32, 33) or antibiotics (29), supplemental oxygen (14, 25, 26), and continuous positive airway pressure or assisted ventilation (27, 32, 33).

#### Study Designs

The following study designs were used in the included preventative research: two randomized controlled trials (33, 34), three counterbalanced quasi-experimental studies (27–29), and six crossover studies of which four were randomized (14, 25, 26, 30) and two were quasi-experimental (31, 32). In most of these studies, the data of equal lasting periods with and without stimulation were compared. The shortest on/off period took 10 min with a total duration of 1 h (26) and the longest four days with a total duration of eight days (28). Kattwinkel et al. (31) stimulated 5 out of 15 min instead of continuous stimulation during the stimulation period. In the controlled randomized trials half of the included infants only received continuous stimulation, which lasted for 7 days (33, 34).

#### Stimulation Systems

Only one study used manual stimulation, which was accomplished by rubbing the extremities of the infant (31). Cardiorespiratory monitoring and HR and respiratory pneumograms were used to detect apnea. All other studies used mechanical ways of continuous stimulation composed of the following: a cuff placed under the upper thorax pulsating 16 ± 4 times/min (27), a 128 Hz vibrating disk attached to the foot (25), two vibrating mattress using exerting a filtered white noise signal of 30–60 Hz with a displacement of 0.01–0.02 (14), respectively, 0.09 mm (26), four water mattresses with varying mean frequencies ranging from 8 to 16 oscillations/min and amplitudes of 1–2.4 mm (28–30, 34), one oscillating air mattress with a frequency of 14–16 oscillations/min (33) and one oscillating and vibrating mattress with a frequency of 5–20 oscillations/min and 8–10 Hz (32). The composition of signals that were recorded varied a lot between the studies. In almost all studies, the HR and oxygen saturation levels were monitored with the aid of a pulse oximeter or cardiorespirography. In some cases thoracic impedance derived by plethysmography (3, 26) or pneumography (30, 31) enabled the detection of ceased breathing effort. Impeded airflow was detected by nasal airflow or temperature sensors (26, 27, 29). There was also a large variation between the studies in thresholds for identifying breathing pauses, bradycardia, and hypoxia. Kesavan et al. (25) counted breathing pauses of 3–5 and >5 s while Svenningsen et al. (32) counted apneas lasting for more than 20 s accompanied by bradycardia and hypoxia. The threshold for bradycardia ranged between <80 and <110 bpm and for oxygen desaturation between <85 and <90%.

#### Effect

Preventative manual stimulation showed a significant decrease in frequency of apnea during the stimulation period (*p* < 0.01). This difference was present during the whole experiment although stimulation was only administered 5 out of every 15 min. All four studies using a vibratory stimulus reported a significant decrease in apneic spells or breathing pauses (14, 25, 26, 32). Three of these studies also showed a significant decrease in amount and/or duration of hypoxic episodes (14, 25, 26). Kesavan et al. (25) reported a significant reduction in amount and duration of bradycardia, and Smith et al. (14) reported only a significant reduction in intensity of bradycardia. The pulsating cuff used in the study of Jirapaet significantly decreased the total amount of apneic episodes during stimulation (27). However, analysis by type of apnea showed that the decrease was only statistically significant for central and mixed apnea. The six studies using oscillating stimulation *via* water and air mattresses showed a more variable effect. Two studies reported a significant decrease in apnea during stimulation (32, 34). Korner et al. (28) showed a decrease in all types of apnea and a significant decrease in apnea combined with bradycardia during stimulation. Despite the positive effect on apnea, one of these studies reported an increase in mean arterial blood pressure of 5 mm Hg during oscillation in four infants and restlessness in 3 of the 12 infants after stimulation (32). The remaining three studies reported no difference in the effects of oscillating mattresses compared with non-oscillating mattresses (14, 28, 33). One of these studies even reported that the frequency of apneas of >10 s increased in five out of six infants and also the frequency of severe bradycardia increased and the mean body temperature decreased with 0.1°C. One infant developed hypothermia and six infants required an increase in incubator temperature (30).

## Discussion

The variation in study designs and the clear division between the studies focusing on termination of apnea and the prevention of apnea led to a separate discussion of the results using a narrative format.

### Termination of Apnea

Animal studies have shown that sensory stimulation is important for the onset of breathing after birth (35–37). Although manual stimulation is recommended in the local and international resuscitation guidelines, its affects on the initiation of breathing have been studied only recently in preterm infants (38, 39). To the best of our knowledge, the effect on termination of apnea has not studied but is the most common method used. However, mechanical tactile stimulation has been evaluated in several studies because it might improve the stimulation technique, lead to a faster response and thus shortens the duration of apnea and reduces the chance of cross-contamination.

Two crossover studies showed that automatic vibratory stimulation of 250 Hz, at either the foot or the thorax, is at least equally effective in terminating apnea compared with manual stimulation (19, 23). Furthermore, both studies showed a decrease in stimulus duration upon termination when using vibrotactile stimulation. However, the response time was not significantly reduced as the nurse had to actuate the mechanical stimulation. In contrast to this, Frank et al. (24) and Camargo et al. (22) used a closed-loop system to study the effect of stimulation on the termination of apnea. The devices were able to terminate 90% of all apneas, but these results were not compared with manual stimulation. A few other articles have described the design of a closed-loop vibratory device (10, 17, 40). However, as far as we are aware there are no published clinical trials that compare automatic mechanical stimulation with manual stimulation.

Despite the fact that manual tactile stimulation is common therapy, the exact neural pathway(s) to the respiratory center remain unclear. It is postulated that tactile stimulation affects respiratory control by activating the brainstem reticular formation causing arousal (41). Ioffe et al. showed that the sleep state of fetal lambs changed following electrical stimulation of somatic nerves (42). The magnitude of the respiratory response differed depending on sleep type and was greatest during REM sleep. However, tactile stimuli can also induce spinal and respiratory responses in infants without resulting in cortical arousal (43, 44).

Furthermore, the effect of mechanical stimulation on the respiratory center is dependent on nerves that are targeted. The skin contains multiple sensory receptors, which are all most sensitive to a specific frequency range (45). The sensitivity of glabrous skin of human adults is highest at 250 Hz (40, 46), which was the frequency chosen in all of our included studies that used vibratory stimulation. However, animal studies have shown that the responsiveness of the immature nervous system to vibration is restricted to lower frequencies in newborns (5–300 Hz) compared with adults (5–1,000 Hz) (47, 48). Lower frequencies applied at the thorax are believed to stimulate intramuscular mechanoreceptors such as muscle spindles and Golgi tendon organs (49, 50). These results imply that the location of stimulation also influences the effect on breathing, depending on the presence of certain receptors.

### Prevention of Apnea

In 1975, Kattwinkel et al. (31) showed that manual tactile stimulation every 5 out of 15 min led to significant less apnea in preterm infants. As this intervention will increase the workload of the nurses, various studies have been conducted to investigate the effect of mechanical stimulation on the prevention of apnea.

Oscillating air- or water mattresses were used most often to stimulate the infants and are believed to mimic the *in utero* environment by activation of the somatic proprioceptors or the cutaneous receptors in the skin. In the first study, Korner et al. (34) showed a significant reduction of apnea associated with the irregular oscillating waterbed. In a second study they showed a decrease in all types of apnea and a significant decrease in apnea combined with bradycardia during stimulation (29). However, another study using the same mattress with regular oscillation (30) has failed to demonstrate significant effects, as a randomized trial in 122 infants (33) and a follow-up study in theophylline treated infants (28). The inability to show positive results may be due to habituation in response to the regular oscillation in the first two studies and by the low incidence of apnea in theophylline treated infants in the latter. However, Jones (30) even reported adverse effects in some of the infants, such as increase of apnea, severe bradycardia and hypoxia.

In response to the oscillation beds, Jirapaet (27) aimed to develop a more suitable, feasible, and cheaper stimulation system to prevent apneic episodes in the form of a pulsating balloon placed under the upper thorax. The balloon pulsated 16 + 4 times/min, similar to the frequency of the oscillation in the first study of Korner et al. (34) and is also believed to provide afferent input to the respiratory center. The amount of central and mixed apneas during stimulation significantly reduced. Despite these positive results, no more research has been conducted on the effects of pulsating stimulation on the prevention of apnea.

Svenningsen et al. (32) conducted a study using an oscillating and vibrating mattress to test the effect of multimodal stimulation and found that infants had less apnea when compared with a normal mattress. Furthermore, longer periods of quiet sleep and shorter periods of active sleep were reported when stimulating the infant. This shift in sleep pattern may be an explanation for the lower frequency of apneic spells. Yet other studies have reported increased periods of quiet sleep without a significant decrease in apneas when stimulating the infant (28).

More recent studies have investigated the effect of vibration as the sole stimulus, which resulted in a significant reduction of apnea or interbreath intervals and a significant reduction in intermitted hypoxia in all cases. Two of the three studies also reported a positive effect on the amount and duration or the intensity of bradycardia.

Kesavan et al. (25) stated that a vibratory stimuli applied to the sole of the foot or palm of the hand activates proprioceptors in the joints, which stabilizes breathing by using the inherent reflexive coupling between limb movements and breathing frequency. This reflex is shown in sleeping adults (51) and in neonatal rabbits (52) during passive motion of the limbs. However, the reason to use a frequency of 128 Hz is not explained in the article. Other studies showed that 80 Hz is the optimal frequency for evocation of movement illusions (53).

Smith et al. (14) and Bloch-Salisbury et al. (26) used mattresses that provided stochastic vibratory stimuli, as they hypothesized that small noisy inputs can stabilize unstable rhythms due to the nonlinear properties of the respiratory oscillator. This hypothesis is extensively explained and substantiated through computational models by Paydarfar and Buerkel (54, 55). Based on previous studies (55, 56), it is postulated that the stimulation in the range of 30–60 Hz might affect the respiratory center *via* somatic or visceral mechanoreceptors in the thorax region. The fact that these receptors can influence the respiratory rhythm is supported by studies that used electrical stimulation to activate the intercostal afferents (57, 58). However, Binks et al. showed that vibration of the thoracic surface could also excite intrapulmonary receptors as it vibrates the lung (59). The stretch receptors in the lung are responsible for inhibition of inspiration following increase in lung volume (60). Furthermore these receptors are believed to act on the airway smooth muscle tone, systemic vascular resistance and HR (61). The last hypothesis is that stochastic resonance directly stimulates gas exchange within lung tissue by mechanical perturbations (14), although this hypothesis has not been substantiated. Yet, experiments in guinea pigs showed that ventilation with added noise improved gas exchange compared with conventional ventilation (62).

It is possible that continuous mechanical stimulation, as is used in all included studies, could negatively influence the sleeping cycles of the infant by causing arousal or increasing the amount of active sleep. However, Bloch-Salisbury et al. (26) showed that on and off switching of the vibrating mattress did not result in significant changes in behavioral state or electroencephalogram power spectrum, suggesting that this form of stimulation does not cause arousal. Although no negative effect on sleep state and other characteristics such as (28, 33) respiration rate, temperature, and emesis were found in studies that used oscillating stimuli for multiple days, it remains unclear whether prolonged continuous stimulation would lead to adverse effects in the infants.

### Limitations

In this systematic review only English articles were considered. Relevant articles found in three databases and additional interesting references were included. By using this methodology it cannot be ruled out that relevant articles are missed. Furthermore the decision to include all modes of stimulation led to a high variety of, i.e., study designs, goals, definitions, measuring methods, and results. These big differences made it very difficult to compare the results.

### Further Implications

In most of the studies, tactile stimulation had a positive effect on the amount of apnea or was able to successfully terminate the apnea, but many important questions remain unanswered. The main issue would be finding out how to stimulate the most optimal pathway to the respiratory center. This means that more research should be performed on the effect of different frequencies, amplitudes, and locations of stimulation on all types of apnea but also to the influence of sleep state, hypoxia, and other environmental effects as well as possible adverse effects such as arousal and habituation.

Closed-loop systems should be used in studies that investigate the effect of stimulation on the termination of apnea with the aim to prove the added value of a direct response. Although continuous stimulation of infants might prevent apnea without causing harm, it may be more beneficial to only stimulate the infant when needed (63). This requires development of algorithms to predict apneic spells or risk of AOP. Two studies proposed algorithmic frameworks that generate predictive warnings, but more research is needed to develop a watertight forecasting system (63, 64).

## Conclusion

In conclusion, it is clear that somatic afferents can influence respiratory center activity. Although manual tactile stimulation is the most common intervention for interruption of apnea, the effectiveness of different techniques were not studied. Mechanical stimulation is believed to improve the current treatment by reducing the risk of cross-contamination and enabling a direct response, but data are scarce. Studies demonstrated that it is possible to terminate apnea with a closed-loop mechanical pulsating or vibrating system and that mechanical vibratory stimulation of 250 Hz is equally effective as manual stimulation in terminating apnea.

Several studies investigated the effect of tactile stimulation on the prevention of apnea. However, there were large variations between the studies in terms of study design, stimulation characteristics and measured outcomes. Although an oscillating mattress was used in six studies, this form of stimulation did not lead to a consistent effect in reducing apnea. Continuous pulsating significantly reduced central and mixed apnea but was only studied once. Different forms of vibrating stimuli were shown to significantly reduce apnea, hypoxia, and bradycardia.

To select the most effective way of stimulation to treat or prevent apnea, more knowledge is required about the neuronal pathways to the brains that are activated by mechanical tactile stimulation, the effect on all types of apnea and the corresponding adverse effects. More studies are needed to confirm whether automated stimulation using a closed loop is more effective than manual stimulation, how and where the automated stimulation should be performed and the potential side effects.

## Author Contributions

SC performed the literature search, selected the studies, and wrote the first draft of the manuscript. AP contributed to selecting the studies and writing an editing the manuscript. All the authors contributed to the final draft of the manuscript by critically reviewing previous manuscripts.

## Conflict of Interest Statement

The authors declare that the research was conducted in the absence of any commercial or financial relationships that could be construed as a potential conflict of interest.

## Abbreviations

AF, nasal airflow; CPAP, continuous positive airway pressure; ECG, electrocardiogram; EEG, electroencephalogram; EMG, electromyogram; GA, gestational age; HR, heart rate; IH, intermittent hypoxia; IPPV, intermittent positive pressure ventilation; NICU, Neonatal Intensive Care Unit; PF, pulse frequency; RR, respiration rate; RM, respiratory movement; SpO_2_, peripheral oxygen saturation.
